# Drift Drives Foraminiferal Community Assembly on a Carbonate Platform

**DOI:** 10.1002/ece3.71604

**Published:** 2025-06-18

**Authors:** Tao Li, Bo Li, Ziya Lin, Wei Xie, Chupeng Yang

**Affiliations:** ^1^ Key Laboratory of Marine Mineral Resources, Ministry of Natural Resources, Guangzhou Marine Geological Survey China Geological Survey Guangzhou China; ^2^ National Engineering Research Center for Gas Hydrate Exploration and Development Guangzhou China; ^3^ School of Marine Sciences/Research Center of Ocean Climate Sun Yat‐Sen University & Southern Marine Science and Engineering Guangdong Laboratory (Zhuhai) Zhuhai China

**Keywords:** benthic foraminifera, community assembly, co‐occurrence network, drift, null and neutral models

## Abstract

Community composition is determined by four processes: drift, selection, dispersal, and speciation. The crucial issue in understanding community assembly is disentangling the relative importance of those processes. However, this issue has not been adequately addressed in benthic foraminiferal communities. A comprehensive study of benthic foraminiferal community composition, co‐occurrence network, and community assembly was conducted on the Xisha carbonate platform. The community composition was determined via the environmental DNA (eDNA) technique. Heavy metals (Co, Cr, Cu, Ni, Pb, V, and Zn), grain size, loss on ignition (LOI), organic carbon, and pH were chosen for environmental measurement. We evaluated the effects of environmental variables on the community composition and the co‐occurrence network, revealing that the former was affected only by organic carbon, whereas the latter was affected by both organic carbon and pH. Null and neutral models demonstrated that foraminiferal community assembly was driven by ecological drift instead of selection. The β‐NTI (a measure of the relative importance of deterministic and stochastic processes) had strong and positive correlations with community β‐diversity (compositional differences between pairs of communities) and network β‐diversity (structural differences between pairs of subnetworks). A conceptual model was offered to explain how heterogeneous selection and stochastic processes interact to affect the two β‐diversities. This study is the first to quantitatively assess the effects of variation in the relative importance of deterministic and stochastic processes on community β‐diversity and network β‐diversity in foraminifera; it provides new insight into the mechanisms underlying β diversity.

## Graphical abstract

We investigate benthic foraminiferal communities on the Xisha carbonate platform via the eDNA technique and conduct a comprehensive study, including community composition, co‐occurrence network, and community assembly. We assess the relative importance of deterministic and stochastic processes in foraminiferal community assembly and find that drift is the main driving force. We hypothesize that community β‐diversity and network β‐diversity increase with the strength of heterogeneous selection and provide a conceptual model to test our hypothesis.

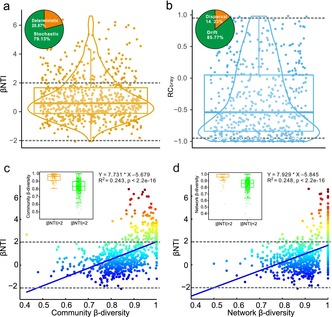

## 1 Introduction

The development of ecological community theory has stimulated interest in the processes or mechanisms that explain community patterns. There are four primary ecological processes that lead to changes in community structure: drift, selection, dispersal, and speciation (Vellend 2010). Drift reflects random changes in relative abundances of species due to birth, death, and reproduction; selection highlights fitness differences (e.g., differences in survival, growth, and reproduction) among different species; dispersal refers to the movement and colonization of a species from one site to another; and speciation indicates the net outcome of extinction and speciation (Zhou and Ning 2017). Drift, dispersal, and speciation are typically classified as stochastic processes, whereas selection is defined as a deterministic process (Zhou and Ning 2017). It is widely accepted that the interactions between deterministic and stochastic processes control community composition (Evans et al. 2017; Vellend et al. 2014).

A long‐standing issue in community ecology is the challenge of disentangling and qualifying the relative importance of deterministic and stochastic processes in shaping community structure (Chase and Myers 2011; Rosindell et al. 2012; Vellend et al. 2014), and many null and neutral models have been extensively cited in the literature (Zhou and Ning 2017). Null models are generated by randomizing a community data matrix or by random sampling from a given distribution function, aiming to obtain predictions if a particular mechanism does not apply (Gotelli and Graves 1996). The beta nearest taxon index (βNTI), a quantitative index for assessing the relative importance of deterministic and stochastic processes, is a widely used null model that has been successfully used to uncover the underlying assembly mechanisms of soil and sediment microbial communities (Jiao et al. 2020; Lu et al. 2022; Stegen et al. 2013; Wang et al. 2021) and microeukaryotic plankton communities (Hou et al. 2020). Neutral models emphasize the crucial role of dispersal limitation, speciation, and drift in community structure (Matthews and Whittaker 2014; Rosindell et al. 2011). Hubbell's neutral model is the most influential model. It assumes that individuals are ecologically equivalent regardless of species identity (Hubbell 2011) and that a dead individual is immediately replaced by another individual: the zero‐sum assumption (Rosindell et al. 2011). Sloan et al. (2006) developed a neutral community model (NCM) to fit the observed abundance–frequency relationship with a beta distribution derived from Hubbell's neutral theory. Sloan's NCM provides strong evidence for the dominance of stochastic processes in the assembly of planktonic bacterial and microeukaryotic communities (Chen et al. 2019; Sun et al. 2021). The combination of null and neutral models provides a comprehensive picture of the community assembly mechanism (e.g., Li et al. 2022; Lu et al. 2022). However, null and neutral models are rarely used for benthos, which hinders our understanding of the processes that govern the assembly of benthic communities.

Benthic foraminifera are among the most abundant marine benthic protozoa (Frontalini et al. 2010), and they play crucial roles in maintaining marine ecosystem function. Like microbial eukaryotes (Bik et al. 2012), some foraminiferal species have a cosmopolitan distribution, while the majority of species seem to be regionally restricted (Gooday and Jorissen 2012). Benthic foraminifera can serve as proxies for changes in benthic macrofaunal community structure because they present a distribution pattern similar to that of benthic macrofauna (Bouchet et al. 2018; Denoyelle et al. 2010; Mojtahid et al. 2008; Włodarska‐Kowalczuk et al. 2013). Importantly, the density and diversity of benthic foraminifera are nearly comparable to those of the entire macrofauna (Denoyelle et al. 2010), which makes benthic foraminifera suitable for addressing broader questions concerning the distribution of marine benthos. Benthic foraminifera are good bioindicators of environmental changes (Włodarska‐Kowalczuk et al. 2013), and their distribution patterns are largely influenced by environmental factors. In shelf and coastal areas, the main influencing factors include temperature, salinity, sediment type, vegetation, and light penetration; on continental slopes, food quantity and quality, sediment type, and topographic features are the main influencing factors; and in deep‐sea areas, the quantity and quality of organic matter and carbonate dissolution are crucial (Gooday and Jorissen 2012; Murray 2006). In addition, their distribution patterns are affected by other factors or processes such as dispersal and biotic interactions (Alve 1999; Murray 2006). A previous study (Li et al. 2022) revealed that environmental conditions and dispersal were the main factors controlling foraminiferal community composition, although the former had a greater effect.

The Xisha carbonate platform is located in the northwestern South China Sea. It is a tropical shallow‐water platform rich in biological resources: corals, fishes, shellfish, marine mammals, seabirds, vegetation, etc. The location and unique ecosystems make this area ideal for investigating ecological evolution and climate change (Liu et al. 2012). A previous morphology‐based survey revealed species diversity and distribution patterns of benthic foraminifera in coral reef areas (Meng et al. 2020), but relatively few studies have concentrated on the distribution of benthic foraminifera on the platform.

In this study, we investigated benthic foraminiferal communities on the Xisha carbonate platform via the eDNA technique and conducted a comprehensive study, including community composition, co‐occurrence network, and community assembly. This study aimed to (1) assess the relative importance of deterministic and stochastic processes in foraminiferal community assembly and (2) uncover correlations between variations in the relative importance of assembly processes and β‐diversity patterns. Wang et al. (2017) noted that selection may be the main driver of the β‐diversity pattern of microbial communities, and later studies (Huber et al. 2020) proved that heterogeneous selection promotes a greater β‐diversity; however, research on their quantitative correlation rather than qualitative description is rare. Here, we hypothesized that community β‐diversity and network β‐diversity increase with the strength of heterogeneous selection.

## 2 Materials and Methods

### 2.1 Study Area and Sampling

**FIGURE 1:**
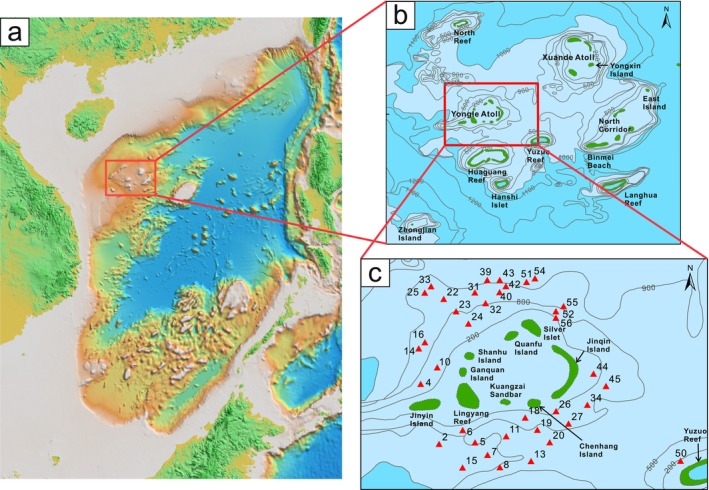
Maps showing the location of the Xisha carbonate platform (Xisha Islands) in the South China Sea (a), the topography of the Xisha carbonate platform (b), and the distribution of sampling sites surrounding the Yongle Atoll (c). The topographic map of the Xisha Islands is redrawn from Zhao (2020). In (a), colors indicate the elevation: The land areas are colored in green and orange and the sea in pink and blue. In (b) and (c), the water depths are shaded with blue, with darker colors representing deeper water.

The Xisha carbonate platform (15°46′−17°08′N, 110°11′−112°54′E) is located in the northwestern part of the South China Sea and southeast of Hainan Island (Figure 1a), covering an area of 5.0 × 10^5^ km^2^. It encompasses more than 40 islands, sandbars, reefs, and shoals. The two largest island groups, the Xuande Atoll and the Yongle Atoll, lie on the east and southwestern sides, respectively (Figure 1b).

The Xisha carbonate platform stands on a submarine terrace of the northern slope, which inclines from northwest to southeast (Xie 1979). The surrounding troughs cut deeply into the seafloor, separating the terrace from the slope (Xie 1979). Influenced by monsoons, seawater flows northeastward in the spring and summer and southwestward in the fall and winter (Shen et al. 2013).

Field work was conducted in the west‐central region of the Xisha carbonate platform in August 2022. Surface sediment samples were collected from 37 sites surrounding the Yongle Atoll via a box sampler (Figure 1c), and the water depth ranged from 280 to 950 m. Only the top 2 cm were sampled, yielding two sets of samples. One set (~50 g) was transferred into sterile plastic tubes for foraminiferal eDNA analyses, and the other was placed in plastic bags (~500 g) for physicochemical analyses. All samples were kept frozen at −20°C until laboratory analyses.

### 2.2 Environmental DNA Extraction, PCR Amplification and High‐Throughput Sequencing

eDNA was extracted from sediment via the FastDNA Spin Kit for Soil (MP bio, USA) according to the manufacturer's instructions, whereas the lysis time was prolonged to 40 min to improve the efficiency of DNA extraction. Three replicate samples were taken at every sampling site for DNA extraction. The hypervariable region of the nuclear 18S rRNA gene (37 + 41f) was amplified via PCR using foraminiferal‐specific primers (forward F1 5′‐AAGGGCACCACAAGAACGC‐3′ and reverse 17–5′‐CGGTCACGTTCGTTGC‐3′) (Frontalini et al. 2020), which produced sequences ranging from 230 to 380 bp. The Illumina PE300 platform was used for sequencing, which was conducted by Shanghai Majorbio Pharm Technology Co. Ltd. The above experimental methods are detailed in Supporting Information.

Following demultiplexing, the DNA fragments were merged via FLASH (version 1.2.11; https://ccb.jhu.edu/software/FLASH/index.shtml) (Magoč and Salzberg 2011). To retain high‐quality data, we used fastp (version 0.20.0; https://github.com/OpenGene/fastp) (Chen et al. 2018) to filter out sequence reads with a mean quality score less than 20 and used the DADA2 plugin (Callahan et al. 2016) within the QIIME 2 pipeline (version 2020.2; http://qiime.org/install/index.html) (Bolyen et al. 2019) to reduce noise. The sequences were clustered into operational taxonomic units (OTUs) with a 95% similarity cutoff (Frontalini et al. 2020) using UPARSE. OTUs were taxonomically assigned via the Basic Local Alignment Search Tool (BLAST) in the Protist Ribosomal Reference (PR2) database (https://github.com/pr2database/pr2database) (Guillou et al. 2012). OTUs were removed if they could not be assigned to foraminifera or if there were fewer than 10 reads. The sequences were rarefied to 2322 per site.

### 2.3 Sediment Physiochemical Property Analysis

The physicochemical parameters included heavy metals, grain size, loss on ignition (LOI), organic carbon, and porewater pH. The sediment samples were dried, ground, and reduced to a fine powder, and heavy metals (Co, Cr, Cu, Ni, Pb, V, and Zn) were measured via an inductively coupled plasma mass spectrometer (ICP‐MS, Agilent 7900, Agilent Technologies Japan Ltd., Japan). The measurement procedures, quality assurance, and quality control of the heavy metals were documented in our previous work (Li et al. 2021). The detection limits were as follows: Co: 0.03 mg kg^−1^, Cr: 0.015 mg kg^−1^, Cu: 0.05 mg kg^−1^, Ni: 0.01 mg kg^−1^, Pb: 0.09 mg kg^−1^, V: 0.06 mg kg^−1^, and Zn: 0.065 mg kg^−1^. Undetected concentrations were replaced by 1/2 of the detection limits. A Mastersizer 3000 laser particle size analyzer (Malvern Instruments Ltd., UK) was used for sediment grain size analysis after the removal of carbonate and organic fractions with HCl and H_2_O_2_. The LOI was measured following the new method recommended by Heiri et al. (2001). The potassium dichromate‐sulfuric acid (K_2_Cr_2_O_7_‐H_2_SO_4_) oxidation method (Bremner and Jenkinson 1960) and the gasometric method (Jones and Kaiteris 1983) were applied to measure organic carbon and calcium carbonate, respectively. The sediments were centrifuged to extract the porewater, and the pH was measured via a Mettler Toledo pH meter (SG78‐FK‐ISM).

To assess anthropogenic contamination in marine sediments, the geo‐accumulation index (*I*_geo_) (Müller 1969) was calculated using the following formula:

(1) $$I_{\mathrm{geo}}=\log_{2}\left(C_{n}/1.5B_{n}\right)$$

where *C*_*n*_ represents the measured concentration of a heavy metal in marine sediments and *B*_*n*_ represents the background level of the heavy metal. The mean element abundance in the upper continental crust (Reimann and de Caritat 1998) serves as the background level, and a coefficient of 1.5 is used because lithogenic effects cause variations in the background concentrations (Müller 1969). The evaluations of sediment contamination are as follows: *I*_geo_ ≤ 0: practically uncontaminated; 0 < *I*_geo_ < 1: uncontaminated to moderately contaminated; 1 < *I*_geo_ < 2: moderately contaminated; 2 < *I*_geo_ < 3: moderately to heavily contaminated; 3 < *I*_geo_ < 4: heavily contaminated; 4 < *I*_geo_ < 5: heavily to extremely contaminated; and *I*_geo_ > 5: extremely contaminated (Müller 1969).

### 2.4 Co‐Occurrence Network Analyses

A partial correlation‐based method called sparse inverse covariance estimation for ecological association inference (SPIEC–EASI) was carried out to generate a co‐occurrence network via the R package *SpiecEasi* with the argument method “glasso”. The SPIEC–EASI can eliminate spurious associations that arise from correlation‐based approaches (Kurtz et al. 2015). To reduce the risk of forming fake correlation, only those OTUs that occurred in at least two samples were included in generating the network. The network was then visualized via the R package *igraph*. Topological properties were described by average degree, average path distance, centralities, density, and transitivity; all were calculated via the package *igraph*. The average degree represents the average number of connections between nodes, and the average path distance represents the average number of steps along the shortest paths for all pairs of nodes. Centralities contain four types of measures: degree, betweenness, closeness, and eigenvector centralities. These centralities are calculated on the basis of node degree, the extent to which a node is located between other pairs of nodes, the distance of a node from all other nodes, and the centrality of the neighbors of a node, respectively (Kolaczyk and Csárdi 2014). Density is the ratio of actual edges to potential edges, and transitivity refers to the proportion of node triples that form triangles (Kolaczyk and Csárdi 2014). The node degree distribution was fitted by a power law distribution using the R package *poweRlaw* (Gillespie 2015). A Python‐based program “robustness” (Iyer et al. 2013) was used to assess network robustness, e.g., network resiliency against targeted attacks (the removal of nodes on the basis of centrality measures) or random attacks (the removal of nodes at random). The boral model (Bayesian ordination and regression analysis), a joint species distribution model developed by Hui (2016), was used to determine whether the correlations between OTUs were due to biotic interactions or environmental filtering. The model was implemented using the R package *boral* (Hui 2018). Differences in the trace of the estimated residual covariance matrix induced by the latent variables were used to quantify the extent to which the co‐occurrence of OTUs was explained by environmental covariates (Hui 2016).

Sample subnetworks were extracted by selecting OTUs that existed in a given sample (Liu et al. 2023). Network β‐diversity was defined as the proportion of shared edges between two networks, varying between 0 (complete overlapping) and 1 (complete non‐overlapping) (Poisot et al. 2012), and was calculated as follows (Carstensen et al. 2014; Poisot et al. 2012):

(2) $$\beta_{w}=\frac{a+b+c}{\left(2a+b+c\right)/2}-1$$

where *a* is the number of shared edges between two networks, *b* is the number of edges unique to the first network, and *c* is the number of edges unique to the second network.

### 2.5 Statistical Analyses

To reveal the characteristics of benthic foraminiferal diversity, α‐ and β‐diversities were calculated among sites via the R package *vegan*, including the ACE index, the Shannon–Wiener index, the Simpson index and the Bray–Curtis dissimilarity. To explore the correlation between foraminiferal OTU composition and physiochemical parameters, multivariate regression tree (MRT) analysis was carried out via the R package *mvpart* (Borcard et al. 2018). MRT analysis forms a tree to visualize clusters of sites; each cluster represents a species assemblage and the corresponding environmental characteristics (De'ath 2002). The responses of OTUs to specific parameters were predicted via random forest via the R package *rfPermute* (Jiao et al. 2018), and the relative importance of OTUs was estimated via the percentage increase in the mean square error (Breiman 2001). The R code for the random forest analysis and significance test is available at GitHub (https://github.com/lyao222lll/sheng‐xin‐xiao‐bai‐yu).

The relationships among the properties of the sample subnetworks, physiochemical parameters, and community α‐diversities were explored via the Mantel test and Pearson's correlation analysis via the R package *vegan*. To explore causal relationships, partial least squares structural equation modeling (PLS–SEM) was performed via the Smart‐PLS 4.1.0.3 program (Hair et al. 2022). The significance of path coefficients was tested by bootstrapping (5000 subsamples). The goodness of fit (*GoF*) was used for an overall measure of model fit for PLS–SEM, which is defined as the geometric mean of the average communality ($\bar{\text{communality}}$) and the average of the variance explained by the model ($\bar{R^{2}}$) (Wetzels et al. 2009):

(3) $$\mathrm{GoF}=\sqrt{\bar{\text{Communality}}\times \bar{R^{2}}}$$

The *GoF* criteria for small, medium, and large effect sizes of the explained variance (*R* ^2^) are as follows: *GoF*_small_ = 0.1, *GoF*_medium_ = 0.25, and *GoF*_large_ = 0.36 (Wetzels et al. 2009).

### 2.6 Analyses of Community Assembly Mechanisms

We performed Sloan's NCM (Sloan et al. 2006) to test the significance of stochastic processes in foraminiferal community assembly. This model suggests that individuals are randomly chosen to lose and then are immediately replaced by immigrants from the metacommunity (source pool of individuals) with a probability of *m* or by the descendants of the local community with a probability of 1–*m* (Sloan et al. 2006). The parameter *m*, a measure of dispersal limitation (Burns et al. 2016), was obtained via the nonlinear least squares method. The overall model fit (*R* ^2^) was assessed by comparing the sum of squares of the residuals and the total sum of squares (Burns et al. 2016). The results are shown as a plot of the predicted frequencies of occurrence of OTUs versus the mean relative abundances of OTUs. OTUs were partitioned above, within, or below 95% confidence intervals of neutral predictions, corresponding to OTUs occurring more frequently, equally frequently, and less frequently than expected by the NCM model (Chen et al. 2019; Lu et al. 2022). The R code for implementation is available from GitHub (https://github.com/Weidong‐Chen‐Microbial‐Ecology/Stochastic‐assembly‐of‐river‐microeukaryotes).

To further explore foraminiferal community assembly, a null model analysis based on βNTI was conducted to assess the relative importance of stochastic versus deterministic processes in the foraminiferal community assembly. Phylogenetic signals were incorporated into the community structure (Zhu and Shen 2021) to quantify the magnitude and direction of the deviations in the observed phylogenetic community turnover from the null model that is generated using randomization procedures (Stegen et al. 2012). The phylogenetic tree of benthic foraminifera was built via IQ‐TREE v2.3.6 (http://www.iqtree.org/) with an ultrafast bootstrap of 1000 replications, and the best model was selected via ModelFinder (Kalyaanamoorthy et al. 2017) on the basis of the Bayesian information criterion (BIC). The phylogenetic tree was then converted to an ultrametric tree via the *chronopl* function of the R package *ape*. The pairwise phylogenetic turnover between communities was calculated as the mean nearest taxon distance metric (βMNTD) via the R package *picante* (Stegen et al. 2012). The βNTI was calculated as the difference between the observed βMNTD and the mean of the null distribution of the βMNTD (after 999 randomizations) normalized by its standard deviation (Stegen et al. 2013, 2012). The Bray–Curtis‐based Raup–Crick metric (RC_bray_) was used to partition stochastic processes. This index measures the degree to which compositional variation among communities differs from the null expectation that results from randomly distributing Bray–Curtis values (Chase et al. 2011; Stegen et al. 2013). The R code for calculating both the βNTI and RC_bray_ is available at GitHub (https://github.com/stegen/Stegen_etal_ISME_2013), and the results were interpreted on the basis of the following criteria (Chase et al. 2011; Stegen et al. 2013; Zhou and Ning 2017):

$$\text{βNTI}\left\{\begin{array}{l} |\text{βNTI}|>2:\text{deterministic processes}\left\{\begin{array}{l} \text{βNTI}>2:\text{heterogeneous selection}\\ \text{βNTI}<–2:\text{homogeneous selection} \end{array}\right.\\ |\text{βNTI}|<2:\text{stochastic processes}\left\{\begin{array}{l} |{\mathrm{RC}}_{\text{bray}}|>0.95:\text{dispersal}\left\{\begin{array}{l} {\mathrm{RC}}_{\text{bray}}>0.95:\text{dispersal limitation}\\ {\mathrm{RC}}_{\text{bray}}<–0.95:\text{homogenizing dispersal} \end{array}\right.\\ |{\mathrm{RC}}_{\text{bray}}|<0.95:\text{ecological drift and diversification} \end{array}\right. \end{array}\right.$$

For a more intuitive description, the βNTI and RC_bray_ are shown in scatter, box, and violin plots via the R package *ggplot2*.

### 2.7 Development of Conceptual Model

A conceptual model was developed to describe how the interaction of selection and stochastic processes changes community β‐diversity and network β‐diversity. For simplicity, only two communities were considered in the model.

## 3 Results

### 3.1 Physiochemical Characteristics of Sediments

The physicochemical characteristics of the 37 sediments are shown in Table S1. Grain size (sand, silt, and clay), pH, CaCO_3_, and LOI had lower variability, whereas organic carbon and heavy metals (Co, Ni, Cu, Zn, V, Cr, and Pb) varied over a relatively larger range. Nevertheless, within most samples, organic carbon was low (< 1%) (Table S1), and the *I*_geo_ values of heavy metals were near or below 0 (Table S1). Accordingly, we inferred that the Xisha carbonate platform was an oligotrophic and lowly contaminated environment.

### 3.2 Composition and Spatial Patterns of Benthic Foraminiferal Communities

The 18S rRNA gene sequencing generated a total of 154,779 high‐quality sequences, ranging from 2332 to 10,451 sequences per sample. These sequences were grouped into 933 OTUs, and their relative abundances at each site and taxonomy assignments are presented in Table S1. The most frequently detected foraminiferal orders included Textulariida (mean 32.7%), Rotaliida (mean 23.9%), Monothalamids X (mean 22.2%), Monothalamids clade BM (mean 5.5%), and Monothalamids clade C (mean 4.7%). Species richness (the ACE index) varied from 7 to 129 (mean 62), the Shannon index varied from 0.341 to 5.565 (mean 4.061), and the Simpson index varied from 0.079 to 0.954 (mean 0.824) (see Table S4).

**FIGURE 2:**
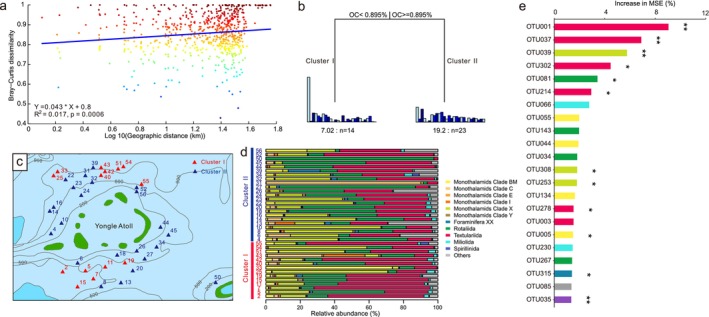
The distance–decay relationship (a), division (b), and spatial distribution (c) of benthic foraminiferal communities, community composition at different sites (d), and the top 22 OTUs in response to organic carbon (e). (a) The distance–decay relationship is revealed by the Spearman's rank correlation between the Bray–Curtis dissimilarity of foraminiferal communities and geographic distance. (b) MRT analysis of the correlations between physiochemical parameters and foraminiferal community composition. Only the most abundant OTUs whose mean relative abundance was > 1% are shown in the bar plots. The numbers under the bars represent the number of samples in two clusters. OC, organic carbon. (c) Sites belonging to clusters I and II are distributed in deeper and shallower water areas, respectively. (d) Different foraminiferal orders are colored differently. (e) The importance of OTUs in response to organic carbon was assessed via a random forest model. A percentage increase in the mean squared error (MSE) is used, and higher MSE% values correspond to greater importance. Bars are colored according to the OTUs in the same way as in (d). Significance levels are as follows: *p < 0.05 and **p < 0.01. MSE, mean squared error.

The Bray–Curtis dissimilarity tended to increase with increasing geographic distance, but the correlation coefficient was small and the slope of the fitting curve was flat (*r* ^2^ = 0.017, slope = 0.043) (Figure 2a), revealing the weak decay of community similarity with geographic distance. Similarly, we observed a weak depth–decay relationship of community similarity (*r* ^2^ = 0.05, slope = 0.037; Figure S1). MRT analysis revealed a simple two‐leaf tree split on the basis of organic carbon (Figure 2b), dividing all the sites into 2 clusters: Cluster I (14 sites) and Cluster II (23 sites). Cluster I included sites in the deeper area where the nutrient content was relatively higher (organic carbon ≥ 0.895%), whereas Cluster II included sites in the shallower area where the nutrient content was lower (organic carbon < 0.895%) (Figure 2c). Cluster I differed from Cluster II mainly in having more Textulariida but fewer Rotaliida (Figure 2d). A random forest was used to predict the response of each OTU to organic carbon, and the most relevant OTUs belonged to Textulariida (e.g., OTU001, OTU037, OTU302, OTU214, and OTU278) and Monothalamids clade X (e.g., OTU039, OTU308, and OTU253) (Figure 2e).

### 3.3 Co‐Occurrence Network and Its Correlation With Environmental Variables

**FIGURE 3:**
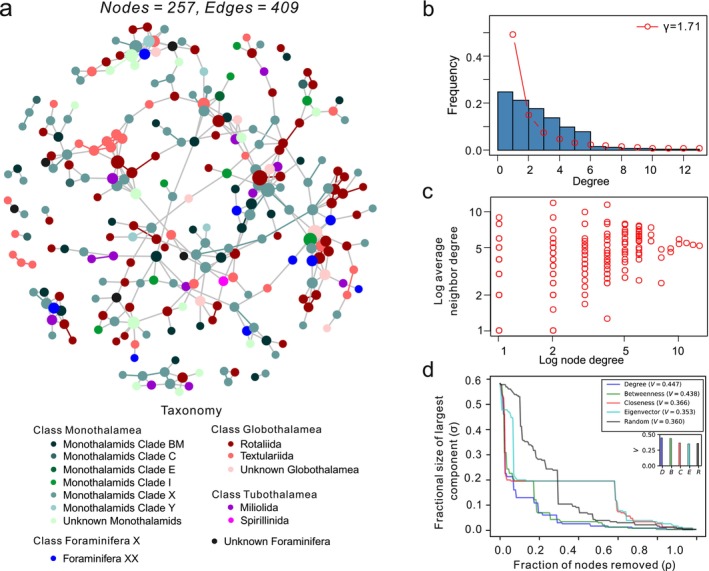
Co‐occurrence network and its properties. (a) Nodes are colored at the order level. Edges that connect two nodes belonging to the same order have the same color with the nodes; otherwise, they are colored gray. The size of each node is proportional to its degree. (b) Plot of the node degree distribution (blue bars) and fitting of a power law distribution to the data (red dotted line). (c) Plot of average neighbor degree versus node degree (log–log scale). (d) Network robustness against targeted attacks based on four kinds of centralities, as well as random attacks. Curves show the fluctuation in the size of the largest component (*σ*) caused by the fraction of nodes removed (*ρ*), and bar charts show the V‐indices, which measure network vulnerability against the attack.

The co‐occurrence network consisted of 257 nodes and 409 edges, with an average degree of 3.18. The co‐occurrence pattern was related to species classification, e.g., OTUs preferentially co‐occurred with those belonging to the same foraminiferal order (Figure 3a). The node degree distribution was roughly fitted by a power law distribution, and the degree exponent (*γ*) was estimated to be 1.71 (Figure 3b). The network could not be a scale‐free network because γ was beyond the scale‐free regime (Barabási 2016). Figure 3c shows that nodes with higher degrees tend to connect with nodes of the same or higher degrees, whereas nodes with lower degrees prefer connecting with nodes of both lower and higher degrees. Figure 3d shows the decrease in the size of the largest component (*σ*) with the increasing fraction of nodes removed (*ρ*). The network was more vulnerable to targeted attacks than to random attacks. Similar impacts were observed among different targeted attacks when *ρ* was less than 0.2; however, as long as *ρ* exceeded 0.2, attacks based on degree and betweenness centralities were more effective at damaging the network than attacks based on the other centralities. This result was reinforced by the V‐index (an index for assessing network vulnerability against the attack, i.e., the removal of nodes), with the former having higher values (Figure 3d).

**FIGURE 4:**
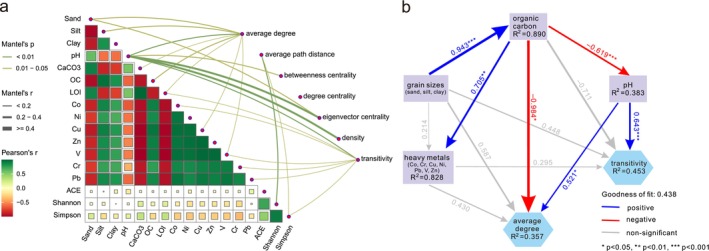
Pearson's correlation analysis versus Mantel test (a) and partial least squares structural equation modeling (PLS–SEM) (b). (a) Pearson's correlation matrix of physiochemical parameters and community α‐diversities is visualized via a heatmap, and the Mantel's test of the topological properties of subnetworks and physiochemical parameters are shown on the right side, where the edge width represents the Mantel's *r* statistic, and edge colors indicate statistically significant values. (b) Path coefficients are shown along paths, and the *R* ^2^ in each box describes the variance of each variable explained by the PLS–SEM results.

The boral model revealed that environmental covariates accounted for approximately 12% of the covariation between OTUs (Figure S2 shows correlations due to environmental covariates and residual correlations), which indicated that the co‐occurrence of OTUs was not mainly determined by environmental filtering. Pearson's correlation revealed that physicochemical parameters were strongly correlated with each other but weakly correlated with community α‐diversities (Figure 4a), indicating that community α‐diversities were poorly predicted from environmental variables. In contrast, the topological properties of each sample subnetwork (see Table S5) were weakly to moderately associated with physicochemical parameters and community α‐diversities (Table S6). The results of Mantel tests revealed that pH was significantly associated with all the physicochemical parameters except the average path distance and degree centrality, whereas the other parameters were mainly associated with the average degree and/or transitivity (Figure 4a). The causal relationship was revealed by the PLS–SEM, which was considered an excellent model because the *GoF* value was larger than the *GoF*_large_ criterion (*GoF* > 0.36) (Figure 4b). This model predicted that changes in transitivity were merely determined by pH, whereas changes in average degree were determined by both organic carbon and pH. Specifically, organic carbon had a negative causal relationship with the average degree, whereas pH had positive causal relationships with the average degree and transitivity. Although the grain size and heavy metals were significantly associated with the average degree and/or transitivity, the causal relationships were not significant.

### 3.4 Deciphering the Assembly Processes of Benthic Foraminiferal Communities

**FIGURE 5:**
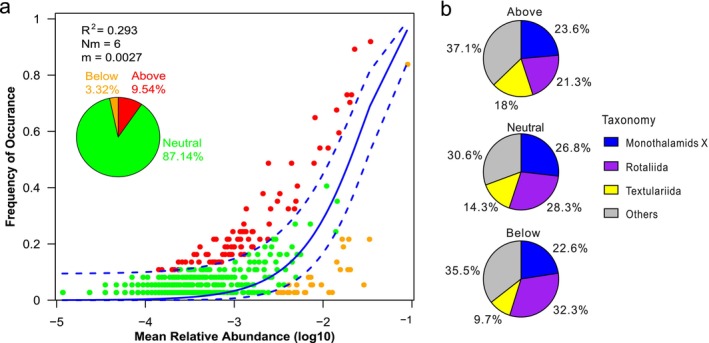
Fit of Sloan's neutral community model of benthic foraminiferal communities. (a) The solid blue line represents the best fit to the neutral model, and the dashed blue lines represent the 95% confidence intervals of the predictions. The predictions of OTUs fall into the above, neutral, and below partitions. They are colored red, orange, and green and correspond to OTUs occurring more frequently, equally frequently, and less frequently than the predicted by the NCM, respectively. *R* ^2^ is the overall fit of the model; Nm is the product of the metacommunity size and migration rate; m is migration rate. (b) Pie charts showing the taxon compositions (Monothalamids X, Textulariida, Rotaliida, and others) in the three partitions.

The NCM fitted well with benthic foraminiferal communities (*R* ^2^ = 0.293). A total of 87.14% of the OTUs occurred equally frequently, 9.54% of the OTUs occurred more frequently, and 3.32% of the OTUs occurred less frequently than expected by the neutral model (Figure 5a). The migration rate parameter *m* was estimated to be 0.0027, revealing that dispersal was limited. More abundant Textulariida and fewer Rotaliida were present in the upper partition than in the lower partition (Figure 5b), indicating that Textulariida had a higher ability of dispersal than Rotaliida did, according to Chen Bolyen et al. (2019).

**FIGURE 6:**
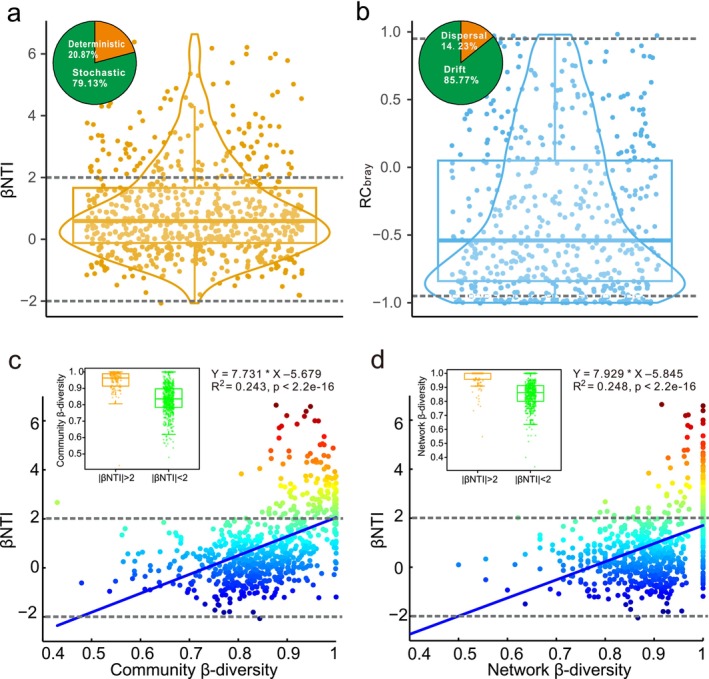
Scatter, box, and violin plots of the βNTI (a) and RC_bray_ (b) and plots of the βNTI versus community β‐diversity (c) and network β‐diversity (d). (a) Deterministic and stochastic processes are identified by the absolute βNTI (|βNTI| > 2, deterministic processes; |βNTI| < 2, stochastic processes). (b) RC_bray_ is calculated under the constraint |βNTI| < 2, partitioning stochastic processes into dispersal and drift (|RC_bray_| > 0.95, dispersal; |RC_bray_| < 0.95, drift). Horizontal dashed lines indicate the significance thresholds of +2 and −2 for the βNTI and those of +0.95 and −0.95 for the RC_bray_.

To further explore the relative contributions of stochastic and deterministic processes to foraminiferal community assembly, βNTI and RC_bray_ were calculated. The βNTI values varied from −2.07 to 6.64, with a median value of 0.59. Approximately 80% of the values were between −2 and 2 (Figure 6a), revealing that foraminiferal community assembly was dominated by stochastic processes. Beyond the range of −2 to 2, nearly all the βNTI values were greater than 2; thus, the deterministic processes were essentially heterogeneous selection (Figure 6a). Under the constraint of βNTI values being greater than −2 and less than 2, the RC_bray_ values varied from −1 to 0.98, with a median value of −0.54. The majority (85%) of the RC_bray_ values were within the range of −0.95 to 0.95 (Figure 6b), indicating that drift had much greater effects than dispersal on benthic foraminiferal communities. The remaining RC_bray_ values were mostly less than −0.95 (Figure 6b), indicating the dominance of homogenizing dispersal over dispersal limitation. In summary, foraminiferal community assembly was dominated by drift (accounting for 67.8% of the total), while the contributions of heterogeneous selection and homogenizing dispersal were relatively small (20.7% and 10.7%, respectively).

### 3.5 Correlations of the βNTI With Community β‐Diversity and Network β‐Diversity

We defined community β‐diversity as the proportional variation in shared species between pairs of communities and network β‐diversity as the proportional variation in shared edges between pairs of sample subnetworks. The correlation analysis revealed that the βNTI was positively correlated with community β‐diversity and network β‐diversity (*r* ^2^ = 0.243 and 0.248, respectively; *p* < 2.2e–16 for both) (see Figure 6c,d), revealing that both community β‐diversity and network β‐diversity increased with increasing βNTI values. Additionally, community β‐diversity and network β‐diversity were significantly greater with an absolute βNTI of < 2 than with an absolute βNTI of > 2 (Figure 6c,d).

### 3.6 Conceptual Model

**FIGURE 7:**
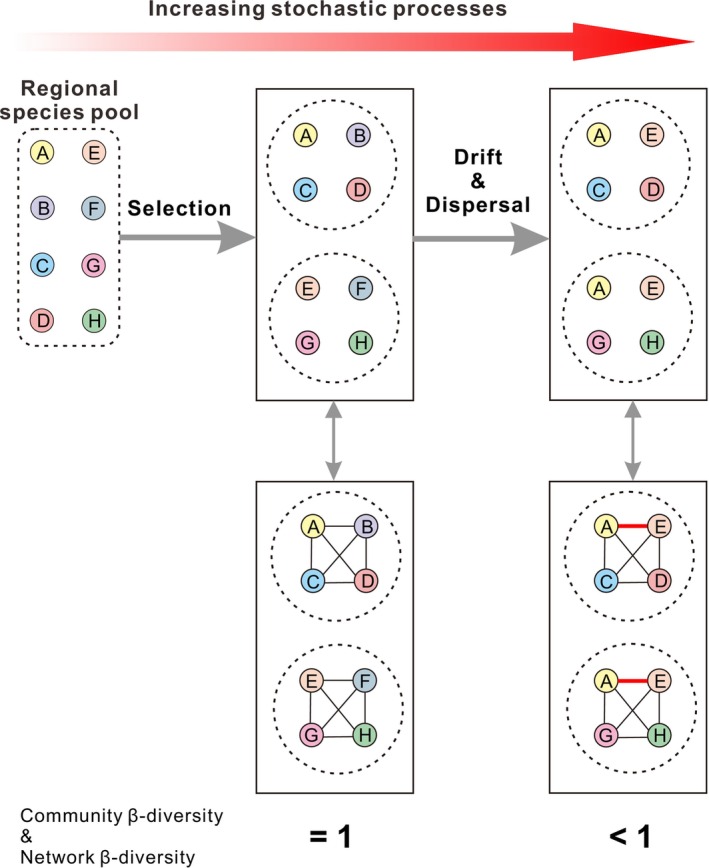
Schematic representation of the differential effects of selection, drift, and dispersal on the community composition and co‐occurrence network. Left: The regional species pool consists of eight species: A, B, C, D, E, F, G, and H. Middle: An extreme example of heterogeneous selection is that species A, B, C, and D constitute one community, whereas species E, F, G, and H constitute another. There are no common links found between the two networks. Right: A general example of drift and dispersal shows that species A and E simultaneously occur in two communities, and the two networks share the same link A–E.

In the conceptual model, all species migrate from a regional species pool, and they have different environmental preferences. Two local communities are imagined under different environmental conditions, and at one extreme, their species compositions are entirely different (i.e., community β‐diversity = 1). Under the influence of heterogeneous selection, species usually co‐occur with those having similar environmental preferences or niches (e.g., links among species A, B, C, and D and links among E, F, G, and H in Figure 7). The result is that the two networks share no common links (i.e., network β‐diversity = 1). Stochastic processes increase the stochasticity of species spatial distributions, resulting in species distribution patterns indistinguishable from those that arise randomly (Jiao et al. 2020). Some species that have different environmental requirements may co‐occur in the same environment (e.g., species A and E in Figure 7), forming a link between them. As a consequence, both community β‐diversity and network β‐diversity decrease.

## 4 Discussion

This study revealed that drift was the primary driver of foraminiferal community assembly on the Xisha carbonate platform. Although environmental factors did not play a crucial role in shaping foraminiferal community composition, organic carbon and pH were the main factors influencing the community composition and co‐occurrence network. A striking finding was that the βNTI was positively correlated with community β‐diversity and network β‐diversity.

### 4.1 Minor Contribution of Environmental Conditions to Foraminiferal Community Assembly

Environmental selection constituted a small proportion (20.7%) of foraminiferal community assembly on the Xisha carbonate platform. This result was quite different from that in our previous study, in which the niche‐based deterministic process (selection) governed the assembly of foraminiferal communities in the bays of the South China Sea (Li et al. 2022). The importance of environmental conditions in controlling foraminiferal community composition appeared to vary across systems (habitats). We compared the environmental properties of the two systems. The environmental factors varied over a wide range in the bays, indicating a highly heterogeneous environment. In contrast, environmental factors did not vary much on the platform, representing a relatively homogeneous environment. In a highly heterogeneous environment, a wide range of environmental factors serve as environmental filters, exerting strong and extensive influences on community composition. In this situation, the signal of stochastic processes can be masked or reduced by strong selection. In a relatively homogeneous environment, however, environmental factors do not impose strong selection, and stochastic processes can obscure the relationships between environmental variables and community composition (Evans et al. 2017). As a consequence, stochastic processes govern the assembly of communities. In summary, a highly heterogeneous environment leads to the dominance of selection, whereas a homogeneous environment facilitates the dominance of stochastic processes.

Organic carbon was a key environmental factor shaping benthic foraminiferal communities. This finding was in agreement with Gooday and Jorissen's (2012) statement that deep‐sea benthic foraminiferal communities are primarily controlled by the organic carbon flux to the seafloor. Benthic foraminiferal communities were divided into two clusters according to organic carbon content. One cluster was associated with a nutrient‐poor and shallower environment, and the other was associated with a nutrient‐rich and deeper environment. A recent study revealed that organic carbon in the Xisha sediments is mainly of autochthonous (marine) origin (Gao et al. 2024) and thus can originate from surface primary production (Altenbach et al. 1999). We conclude that food inputs derived from surface primary production are crucial for the distribution patterns of benthic foraminifera. In addition, organic carbon primarily affected Textulariida and Monothalamids clade X (Figure 2e), revealing that these taxa were more sensitive to changes in organic carbon than were the others. Other parameters, such as heavy metals, grain size, LOI, and pH, played minimal roles in shaping community composition due to their low concentrations. Environmental variables may act as limiting factors when they exceed the tolerance level of a species (Murray 2001, 2006), but at much lower levels, their influences are no longer important (Gooday and Jorissen 2012). Furthermore, we examined the correlation between the co‐occurrence network and environmental factors to evaluate the effects of environmental factors on community assembly. Our analyses revealed that organic carbon and pH were the two main drivers of the co‐occurrence patterns (Figure 4b). Specifically, the average degree showed negative and positive causal relationships with organic carbon and pH, respectively, indicating that benthic foraminifera are more connected under lower organic carbon conditions and/or at higher pH values. A positive causal relationship was observed between pH and transitivity. Transitivity provides a measure of the level of clustering (Barabási 2016), and high transitivity indicates that the network contains communities where nodes are densely connected to each other (Yu and Sun 2024). From this point of view, benthic foraminifera are densely connected at a relatively high pH, which is likely because a relatively low pH is harmful to benthic foraminifera, e.g., erosion of shells. However, the joint species distribution modeling result revealed that only a small proportion of the co‐occurrence of OTUs was explained by environmental filtering, whereas the majority may be due to biotic interactions.

Only some of the environmental parameters were considered in this study, while other parameters, such as temperature, salinity, and pressure, should be further assessed. CTD (conductivity, temperature, and depth) measurements in the Xisha area revealed that temperature varied over a large range (~10°C) while salinity was relatively uniform in deep‐water areas (water depth > 200 m) (Figure S3). We believe that temperature may be a limiting factor for benthic foraminifera, but the effect of salinity is relatively small. Furthermore, pressure changes with water depth and their effects should be similar, whereas water depth does not have a significant effect because of the weak depth–decay relationship of community similarity.

### 4.2 Foraminiferal Community Assembly Mainly Shaped by Stochastic Processes

Stochastic processes overwhelmed the influence of selection involving niche partitioning (Chust et al. 2013), especially drift, which governed foraminiferal community assembly (Figure 6a,b). This result was consistent with previous studies showing that the influence of selection can be overridden by drift when selection is weak and the local community size is small (Chase and Myers 2011; Vellend 2010). Although the sizes of benthic foraminiferal communities are typically large, many taxa are rare (in this study, rare taxa accounted for nearly 40% of the OTUs). The abundance of rare taxa highlights the importance of drift in determining community composition because rare taxa are more easily affected by drift than are common taxa (Nemergut et al. 2013). Additionally, limited dispersal (as discussed later) may promote convergence during adaptation to similar environments, i.e., ecological equivalence (Hubbell 2006; Vellend 2016), thus amplifying the effect of drift (Hanson et al. 2012; Stegen et al. 2013).

Dispersal had a minor role in foraminiferal community assembly. The NCM predicted that the immigration rate was very low (*m* = 0.0027), indicating that the dispersal of benthic foraminifera was limited. This finding was consistent with our previous study (Li et al. 2022). The reasons are as follows. First, benthic foraminifera cannot swim, and passive transport of individuals (at all life stages) is the primary dispersal mechanism (Alve 1999). However, this type of dispersal is not efficient because benthic foraminifera are not easily suspended in seawater (Alve 1999). Second, the Xisha carbonate platform is isolated (Zhao 2020), and the surrounding steep slopes may hinder the migration of benthic foraminifera. Finally, the dispersal and colonization of benthic foraminifera are determined not only by the dispersal rate but also by food availability (Alve 1999). The Xisha carbonate platform is an oligotrophic environment, and food limitations may restrict the colonization of benthic foraminifera. The weak distance–decay relationship (Figure 2a) may indicate that dispersal was high (Hanson et al. 2012), which seemed contrary to our finding of limited dispersal. However, the distance–decay relationship is simultaneously influenced by species sorting, dispersal, and drift (Jiao et al. 2020). It is difficult to assess the direct effect of dispersal on the distance–decay relationship. Additionally, the NCM predicted that Textulariida were better dispersers than Rotaliida were, which may account for the variations in the distribution of the two taxa, according to Gooday and Jorissen (2012).

### 4.3 Community β‐Diversity and Network β‐Diversity Affected by the Strength of Heterogeneous Selection

Previous studies have shown that the βNTI is significantly associated with variation in abiotic and biotic parameters (e.g., Jiao et al. 2022; Stegen et al. 2012; Tripathi et al. 2018), and their relationship can be used to infer the effects of abiotic and biotic parameters on variation in the relative importance of assembly processes (Tripathi et al. 2018). Here, we further inferred the effects of variation in the relative importance of assembly processes on β‐diversity patterns. Because the βNTI‐based null model is designed to detect differences in community composition caused by selection, variation in the magnitude of the βNTI is driven by deterministic processes (Tripathi et al. 2018). We assumed that the relative importance of selection increased with increasing βNTI values (in this case, βNTI > −2; the opposite situation could be expected when βNTI < −2). Interestingly, the βNTI was strongly and positively related to community β‐diversity and network β‐diversity (Figure 6). As the relative importance of heterogeneous selection increased, community β‐diversity and network β‐diversity tended to increase. This finding was consistent with the hypothesis that community β‐diversity and network β‐diversity increase with the strength of heterogeneous selection.

Our hypothesis can be explained further by the conceptual model. In this model, the strength of heterogeneous selection is weakened by stochastic processes, resulting in decreases in community β‐diversity and network β‐diversity. However, it is worth noting that the interactions between different processes are complicated (e.g., Evans et al. 2017; Stegen et al. 2013), and the outcome may vary under different circumstances. Unfortunately, our hypothesis could not be tested in our previous study (Li et al. 2022) because there was no index designed to assess variation in the relative importance of community assembly processes based on morphological data. The correlations of the βNTI with community β‐diversity and network β‐diversity were established under weak selection and limited dispersal. Such correlations should be examined across a broad range of systems, especially those with different environmental conditions and dispersal rates.

## 5 Conclusion

An environmental survey reveals that the Xisha carbonate platform is slightly polluted by heavy metals. The eDNA survey reveals that Textulariida, Rotaliida, Monothalamids clade X, Monothalamids clade BM, and Monothalamids clade C dominate the foraminiferal communities. Organic carbon is the main environmental factor influencing the community composition and the co‐occurrence network, and the latter is also affected by pH. Null and neutral models reveal that foraminiferal community assembly on the platform is driven by drift, whereas the contributions of selection and dispersal are relatively small. The βNTI is strongly and positively correlated with community β‐diversity and network β‐diversity, indicating that the two β‐diversities are driven by heterogeneous selection. Furthermore, we find that foraminiferal community assembly on the platform differs from that in the bays of the South China Sea and hence infer that the community assembly mechanisms may depend on systems (habitats).

Given that benthic foraminiferal communities on the Xisha carbonate platform are highly dependent on stochastic processes and are not determined by environmental conditions, it is difficult to predict the future trend of changes in the benthic foraminiferal community (or even the whole benthic community) living there.

## Author Contributions

**Tao Li:** conceptualization (lead), formal analysis (lead), funding acquisition (lead), methodology (lead), project administration (lead), writing – original draft (lead), writing – review and editing (lead). **Bo Li:** data curation (supporting), investigation (lead), writing – original draft (supporting). **Ziya Lin:** data curation (supporting), writing – original draft (supporting). **Wei Xie:** data curation (lead), formal analysis (supporting), writing – original draft (supporting), writing – review and editing (supporting). **Chupeng Yang:** investigation (supporting), writing – original draft (supporting).

## Conflicts of Interest

The authors declare no conflicts of interest.

## Supporting information


Appendix S1.

Appendix S2.

## Data Availability

The DNA sequences were deposited in GenBank (https://www.ncbi.nlm.nih.gov/) under accession number PRJNA937323.

## References

[ece371604-bib-0001] Altenbach, A. V. , U. Pflaumann , R. Schiebel , et al. 1999. “Scaling Percentages and Distributional Patterns of Benthic Foraminifera With Flux Rates of Organic Carbon.” Journal of Foraminiferal Research 29, no. 3: 173–185.

[ece371604-bib-0002] Alve, E. 1999. “Colonization of New Habitats by Benthic Foraminifera: A Review.” Earth‐Science Reviews 46: 167–185. 10.1016/S0012-8252(99)00016-1.

[ece371604-bib-0003] Barabási, A.‐L. 2016. Network Science. Cambridge University Press.

[ece371604-bib-0004] Bik, H. M. , W. Sung , P. D. Ley , et al. 2012. “Metagenetic Community Analysis of Microbial Eukaryotes Illuminates Biogeographic Patterns in Deep‐Sea and Shallow Water Sediments.” Molecular Ecology 21: 1048–1059. 10.1111/j.1365-294X.2011.05297.x. 21985648 PMC3261328

[ece371604-bib-0005] Bolyen, E. , J. R. Rideout , M. R. Dillon , et al. 2019. “Reproducible, Interactive, Scalable and Extensible Microbiome Data Science Using QIIME 2.” Nature Methods 37, no. 8: 852–857. 10.7287/peerj.preprints.27295v2. PMC7015180 31341288

[ece371604-bib-0006] Borcard, D. , F. Gillet , and P. Legendre . 2018. Numerical Ecology With R. 2nd ed. Springer International Publishing AG.

[ece371604-bib-0007] Bouchet, V. M. P. , R. J. Telford , B. Rygg , E. Oug , and E. Alve . 2018. “Can Benthic Foraminifera Serve as Proxies for Changes in Benthic Macrofaunal Community Structure? Implications for the Definition of Reference Conditions.” Marine Environmental Research 137: 24–36. 10.1016/j.marenvres.2018.02.023. 29503108

[ece371604-bib-0008] Breiman, L. 2001. “Random Forests.” Machine Learning 45, no. 1: 5–32. 10.1023/A:1010933404324.

[ece371604-bib-0009] Bremner, J. M. , and D. S. Jenkinson . 1960. “Determination of Organic Carbon in Soil: I. Oxidation by Dichromate of Organic Matter in Soil and Plant Materials.” Journal of Soil Science 11: 394–402. 10.1111/j.1365-2389.1960.tb01093.x.

[ece371604-bib-0010] Burns, A. R. , W. Z. Stephens , K. Stagaman , et al. 2016. “Contribution of Neutral Processes to the Assembly of Gut Microbial Communities in the Zebrafish Over Host Development.” ISME Journal 10: 655–664. 10.1038/ismej.2015.142. 26296066 PMC4817674

[ece371604-bib-0011] Callahan, B. J. , P. J. McMurdie , M. J. Rosen , A. W. Han , A. J. A. Johnson , and S. P. Holmes . 2016. “DADA2: High‐Resolution Sample Inference From Illumina Amplicon Data.” Nature Methods 13: 581–583. 10.1038/nmeth.3869. 27214047 PMC4927377

[ece371604-bib-0012] Carstensen, D. W. , M. Sabatino , K. Trøjelsgaard , and L. P. C. Morellato . 2014. “Beta Diversity of Plant‐Pollinator Networks and the Spatial Turnover of Pairwise Interactions.” PLoS One 9, no. 11: e112903. 10.1371/journal.pone.0112903. 25384058 PMC4226610

[ece371604-bib-0013] Chase, J. M. , N. J. B. Kraft , K. G. Smith , M. Vellend , and B. D. Inouye . 2011. “Using Null Models to Disentangle Variation in Community Dissimilarity From Variation in α‐Diversity.” Ecosphere 2, no. 2: art24. 10.1890/ES10-00117.1.

[ece371604-bib-0014] Chase, J. M. , and J. A. Myers . 2011. “Disentangling the Importance of Ecological Niches From Stochastic Processes Across Scales.” Philosophical Transactions of the Royal Society, B: Biological Sciences 366: 2351–2363. 10.1098/rstb.2011.0063. PMC3130433 21768151

[ece371604-bib-0015] Chen, S. , Y. Zhou , Y. Chen , and J. Gu . 2018. “Fastp: An Ultra‐Fast All‐in‐One FASTQ Preprocessor.” Bioinformatics 34: i884–i890. 10.1093/bioinformatics/bty560. 30423086 PMC6129281

[ece371604-bib-0016] Chen, W. , K. Ren , A. Isabwe , H. Chen , M. Liu , and J. Yang . 2019. “Stochastic Processes Shape Microeukaryotic Community Assembly in a Subtropical River Across Wet and Dry Seasons.” Microbiome 7: 138. 10.1186/s40168-019-0749-8. 31640783 PMC6806580

[ece371604-bib-0017] Chust, G. , X. Irigoien , J. Chave , and R. P. Harris . 2013. “Latitudinal Phytoplankton Distribution and the Neutral Theory of Biodiversity.” Global Ecology and Biogeography 22: 531–543. 10.1111/geb.12016.

[ece371604-bib-0018] De'ath, G. 2002. “Multivariate Regression Trees: A New Technique for Modeling Species‐Environment Relationships.” Ecology 83, no. 4: 1105–1117. 10.2307/3071917.

[ece371604-bib-0019] Denoyelle, M. , F. J. Jorissen , D. Martin , F. Galgani , and J. Miné . 2010. “Comparison of Benthic Foraminifera and Macrofaunal Indicators of the Impact of Oil‐Based Drill Mud Disposal.” Marine Pollution Bulletin 60: 2007–2021. 10.1016/j.marpolbul.2010.07.024. 20825954

[ece371604-bib-0021] Evans, S. , J. B. Martiny , and S. D. Allison . 2017. “Effects of Dispersal and Selection on Stochastic Assembly in Microbial Communities.” ISME Journal 11: 176–185. 10.1038/ismej.2016.96. 27494293 PMC5315486

[ece371604-bib-0022] Frontalini, F. , R. Coccioni , and C. Bucci . 2010. “Benthic Foraminiferal Assemblages and Trace Element Contents From the Lagoons of Orbetello and Lesina.” Environmental Monitoring and Assessment 170: 245–260. 10.1007/s10661-009-1229-6. 19911292

[ece371604-bib-0023] Frontalini, F. , T. Cordier , E. Balassi , et al. 2020. “Benthic Foraminiferal Metabarcoding and Morphology‐Based Assessment Around Three Offshore Gas Platforms: Congruence and Complementarity.” Environment International 144: 106049. 10.1016/j.envint.2020.106049. 32835923

[ece371604-bib-0024] Gao, J. , K. Yu , S. Xu , et al. 2024. “Content and Source Analysis of Organic Carbon in the Outer Slope Sediments of the Yongle Atoll, Xisha Islands.” Journal of Tropical Oceanography 43: 131–145 (in Chinese with English abstract). 10.11978/2023112.

[ece371604-bib-0025] Gillespie, C. S. 2015. “Fitting Heavy Tailed Distributions: The poweRlaw Package.” Journal of Statistical Software 64, no. 2: 1–16. 10.18637/jss.v064.i02.

[ece371604-bib-0026] Gooday, A. J. , and F. J. Jorissen . 2012. “Benthic Foraminiferal Biogeography: Controls on Global Distribution Patterns in Deep‐Water Settings.” Annual Review of Marine Science 4: 237–262. 10.1146/annurev-marine-120709-142737. 22457975

[ece371604-bib-0027] Gotelli, N. J. , and G. R. Graves . 1996. Null Models in Ecology. Smithsonian Institution Press.

[ece371604-bib-0028] Guillou, L. , D. Bachar , S. Audic , et al. 2012. “The Protist Ribosomal Reference Database (PR2): A Catalog of Unicellular Eukaryote Small Sub‐Unit rRNA Sequences With Curated Taxonomy.” Nucleic Acids Research 41: D597–D604. 10.1093/nar/gks1160. 23193267 PMC3531120

[ece371604-bib-0029] Hair, J. F. , G. T. M. Hult , C. M. Ringle , and M. Sarstedt . 2022. A Primer on Partial Least Squares Structural Equation Modeling (PLS‐SEM). 3rd ed. Sage.

[ece371604-bib-0030] Hanson, C. A. , J. A. Fuhrman , M. C. Horner‐Devine , and J. B. H. Martiny . 2012. “Beyond Biogeographic Patterns: Processes Shaping the Microbial Landscape.” Nature Reviews Microbiology 10: 497–506. 10.1038/nrmicro2795. 22580365

[ece371604-bib-0031] Heiri, O. , A. F. Lotter , and G. Lemcke . 2001. “Loss on Ignition as a Method for Estimating Organic and Carbonate Content in Sediments: Reproducibility and Comparability of Results.” Journal of Paleolimnology 25: 101–110. 10.1023/A:1008119611481.

[ece371604-bib-0032] Hou, F. , H. Zhang , W. Xie , X. Zhou , X. Zhu , and D. Zhang . 2020. “Co‐Occurrence Patterns and Assembly Processes of Microeukaryotic Communities in an Early‐Spring Diatom Bloom.” Science of the Total Environment 711: 134624. 10.1016/j.scitotenv.2019.134624. 31818596

[ece371604-bib-0033] Hubbell, S. P. 2006. “Neutral Theory and the Evolution of Ecological Equivalence.” Ecology 87, no. 6: 1387–1398. 10.1890/0012-9658(2006)87[1387:NTATEO]2.0.CO;2. 16869413

[ece371604-bib-0034] Hubbell, S. P. 2011. The Unified Neutral Theory of Biodiversity and Biogeography. Princeton University Press. 10.1016/j.tree.2011.03.024 21561679

[ece371604-bib-0035] Huber, P. , S. Metz , F. Unrein , G. Mayora , H. Sarmento , and M. Devercelli . 2020. “Environmental Heterogeneity Determines the Ecological Processes That Govern Bacterial Metacommunity Assembly in a Floodplain River System.” ISME Journal 14: 2951–2966. 10.1038/s41396-020-0723-2. 32719401 PMC7784992

[ece371604-bib-0036] Hui, F. K. 2016. “Boral—Bayesian Ordination and Regression Analysis of Multivariate Abundance Data in R.” Methods in Ecology and Evolution 7: 744–750. 10.1111/2041-210X.12514.

[ece371604-bib-0037] Hui, F. K. 2018. “boral: Bayesian Ordination and Regression Analysis. Version 1.7.” Accessed 1 March 2025. http://CRAN.R‐project.org/package=boral.

[ece371604-bib-0038] Iyer, S. , T. Killingback , B. Sundaram , and Z. Wang . 2013. “Attack Robustness and Centrality of Complex Networks.” PLoS One 8: e59613. 10.1371/journal.pone.0059613. 23565156 PMC3615130

[ece371604-bib-0039] Jiao, S. , W. Chen , J. Wang , et al. 2018. “Soil Microbiomes With Distinct Assemblies Through Vertical Soil Profiles Drive the Cycling of Multiple Nutrients in Reforested Ecosystems.” Microbiome 6: 146. 10.1186/s40168-018-0526-0. 30131068 PMC6104017

[ece371604-bib-0040] Jiao, S. , H. Chu , B. Zhang , X. Wei , W. Chen , and G. Wei . 2022. “Linking Soil Fungi to Bacterial Community Assembly in Arid Ecosystems.” iMeta 1: e2. 10.1002/imt2.2. 38867731 PMC10989902

[ece371604-bib-0041] Jiao, S. , Y. Yang , Y. Xu , J. Zhang , and Y. Lu . 2020. “Balance Between Community Assembly Processes Mediates Species Coexistence in Agricultural Soil Microbiomes Across Eastern China.” ISME Journal 14: 202–216. 10.1038/s41396-019-0522-9. 31611655 PMC6908645

[ece371604-bib-0042] Jones, G. A. , and P. Kaiteris . 1983. “A Vacuum‐Gasometric Technique for Rapid and Precise Analysis of Calcium Carbonate in Sediments and Soils.” Journal of Sedimentary Research 53: 655–660. 10.1306/212F825B-2B24-11D7-8648000102C1865D.

[ece371604-bib-0043] Kalyaanamoorthy, S. , B. Q. Minh , T. K. F. Wong , A. von Haeseler , and L. S. Jermiin . 2017. “ModelFinder: Fast Model Selection for Accurate Phylogenetic Estimates.” Nature Methods 14: 587–589. 10.1038/nmeth.4285. 28481363 PMC5453245

[ece371604-bib-0044] Kolaczyk, E. D. , and G. Csárdi . 2014. Statistical Analysis of Network Data With R. Springer.

[ece371604-bib-0045] Kurtz, Z. D. , C. L. Müller , E. R. Miraldi , D. R. Littman , M. J. Blaser , and R. A. Bonneau . 2015. “Sparse and Compositionally Robust Inference of Microbial Ecological Networks.” PLoS Computational Biology 11, no. 5: e1004226. 10.1371/journal.pcbi.1004226. 25950956 PMC4423992

[ece371604-bib-0046] Li, T. , G. Cai , M. Zhang , S. Li , and X. Nie . 2021. “The Response of Benthic Foraminifera to Heavy Metals and Grain Sizes: A Case Study From Hainan Island, China.” Marine Pollution Bulletin 167: 112328. 10.1016/j.marpolbul.2021.112328. 33852988

[ece371604-bib-0047] Li, T. , M. Zhang , B. Li , G. Cai , S. Li , and X. Nie . 2022. “Co‐Occurrence Patterns and Community Assembly Mechanisms of Benthic Foraminiferal Communities in South Chinese Bays.” Ecological Indicators 144: 109489. 10.1016/j.ecolind.2022.109489.

[ece371604-bib-0048] Liu, C. , C. Li , Y. Jiang , R. J. Zeng , M. Yao , and X. Li . 2023. “A Guide for Comparing Microbial Co‐Occurrence Networks.” iMeta 2: e71. 10.1002/imt2.71. 38868345 PMC10989802

[ece371604-bib-0049] Liu, X. , C. Lou , L. Xu , and L. Sun . 2012. “Distribution and Bioavailability of Cadmium in Ornithogenic Coral‐Sand Sediments of the Xisha Archipelago, South China Sea.” Environmental Pollution 168: 151–160. 10.1016/j.envpol.2012.04.018. 22610039

[ece371604-bib-0050] Lu, M. , X. Wang , H. Li , et al. 2022. “Microbial Community Assembly and Co‐Occurrence Relationship in Sediments of the River‐Dominated Estuary and the Adjacent Shelf in the Wet Season.” Environmental Pollution 308: 119572. 10.1016/j.envpol.2022.119572. 35661808

[ece371604-bib-0051] Magoč, T. , and S. L. Salzberg . 2011. “FLASH: Fast Length Adjustment of Short Reads to Improve Genome Assemblies.” Bioinformatics 27, no. 21: 2957–2963. 10.1093/bioinformatics/btr507. 21903629 PMC3198573

[ece371604-bib-0052] Matthews, T. J. , and R. J. Whittaker . 2014. “Neutral Theory and the Species Abundance Distribution: Recent Developments and Prospects for Unifying Niche and Neutral Perspectives.” Ecology and Evolution 4: 2263–2277. 10.1002/ece3.1092. 25360266 PMC4201439

[ece371604-bib-0053] Meng, M. , K. Yu , P. Hallock , and G. Qin . 2020. “Distribution of Recent Foraminifera as Depositional Indicators in Yongle Atoll, Xisha Islands, South China Sea.” Marine Micropaleontology 158: 101880. 10.1016/j.marmicro.2020.101880.

[ece371604-bib-0054] Mojtahid, M. , F. Jorissen , and T. H. Pearson . 2008. “Comparison of Benthic Foraminiferal and Macrofaunal Responses to Organic Pollution in the Firth of Clyde (Scotland).” Marine Pollution Bulletin 56: 42–76. 10.1016/j.marpolbul.2007.08.018. 18054967

[ece371604-bib-0055] Müller, G. 1969. “Index of Geoaccumulation in Sediments of the Rhine River.” Geographical Journal 2, no. 3: 108–118.

[ece371604-bib-0056] Murray, J. W. 2001. “The Niche of Benthic Foraminifera, Critical Thresholds and Proxies.” Marine Micropaleontology 41: 1–7. 10.1016/S0377-8398(00)00057-8.

[ece371604-bib-0057] Murray, J. W. 2006. Ecology and Applications of Benthic Foraminifera. Cambridge University Press.

[ece371604-bib-0058] Nemergut, D. R. , S. K. Schmidt , T. Fukami , et al. 2013. “Patterns and Processes of Microbial Community Assembly.” Microbiology and Molecular Biology Reviews 77, no. 3: 342–356. 10.1128/MMBR.00051-12. 24006468 PMC3811611

[ece371604-bib-0059] Poisot, T. , E. Canard , D. Mouillot , N. Mouquet , and D. Gravel . 2012. “The Dissimilarity of Species Interaction Networks.” Ecology Letters 15: 1353–1361. 10.1111/ele.12002. 22994257

[ece371604-bib-0060] Reimann, C. , and P. de Caritat . 1998. Chemical Elements in the Environment: Factsheets for the Geochemist and Environmental Scientist. Springer‐Verlag.

[ece371604-bib-0061] Rosindell, J. , S. P. Hubbell , and R. S. Etienne . 2011. “The Unified Neutral Theory of Biodiversity and Biogeography at Age Ten.” Trends in Ecology & Evolution 26, no. 7: 340–348. 10.1016/j.tree.2011.03.024. 21561679

[ece371604-bib-0062] Rosindell, J. , S. P. Hubbell , F. He , L. J. Harmon , and R. S. Etienne . 2012. “The Case for Ecological Neutral Theory.” Trends in Ecology & Evolution 27: 203–208. 10.1016/j.tree.2012.01.004. 22341498

[ece371604-bib-0063] Shen, J. , H. Yang , Y. Wang , F. Fu , and N. Zhao . 2013. “Coral Community Dynamics and Shallow‐Water Carbonate Deposition of the Reef‐Flat Around Yongxing Island, the Xisha Islands.” Science China Earth Sciences 56: 1471–1486. 10.1007/s11430-013-4677-3.

[ece371604-bib-0064] Sloan, W. T. , M. Lunn , S. Woodcock , I. M. Head , S. Nee , and T. P. Curtis . 2006. “Quantifying the Roles of Immigration and Chance in Shaping Prokaryote Community Structure.” Environmental Microbiology 8: 732–740. 10.1111/j.1462-2920.2005.00956.x. 16584484

[ece371604-bib-0065] Stegen, J. C. , X. Lin , J. K. Fredrickson , et al. 2013. “Quantifying Community Assembly Processes and Identifying Features That Impose Them.” ISME Journal 7, no. 11: 11. 10.1038/ismej.2013.93. PMC3806266 23739053

[ece371604-bib-0066] Stegen, J. C. , X. Lin , A. E. Konopka , and J. K. Fredrickson . 2012. “Stochastic and Deterministic Assembly Processes in Subsurface Microbial Communities.” ISME Journal 6: 1653–1664. 10.1038/ismej.2012.22. 22456445 PMC3498916

[ece371604-bib-0067] Sun, H. , H. Baozhu , G. He , et al. 2021. “Assembly Processes and Co‐Occurrence Relationships in the Bacterioplankton Communities of a Large River System.” Ecological Indicators 126: 107643. 10.1016/j.ecolind.2021.107643.

[ece371604-bib-0068] Tripathi, B. M. , J. C. Stegen , M. Kim , K. Dong , J. M. Adams , and Y. K. Lee . 2018. “Soil pH Mediates the Balance Between Stochastic and Deterministic Assembly of Bacteria.” ISME Journal 12: 1072–1083. 10.1038/s41396-018-0082-4. 29515169 PMC5864241

[ece371604-bib-0069] Vellend, M. 2010. “Conceptual Synthesis in Community Ecology.” Quarterly Review of Biology 85, no. 2: 183–206. 10.1086/652373. 20565040

[ece371604-bib-0070] Vellend, M. 2016. The Theory of Ecological Communities. Princeton University Press.

[ece371604-bib-0071] Vellend, M. , D. S. Srivastava , K. M. Anderson , et al. 2014. “Assessing the Relative Importance of Neutral Stochasticity in Ecological Communities.” Oikos 123: 1420–1430. 10.1111/oik.01493.

[ece371604-bib-0072] Wang, J. , L. Wang , W. Hu , et al. 2021. “Assembly Processes and Source Tracking of Planktonic and Benthic Bacterial Communities in the Yellow River Estuary.” Environmental Microbiology 23, no. 5: 2578–2591. 10.1111/1462-2920.15480. 33754415

[ece371604-bib-0073] Wang, X. , X. Lü , J. Yao , et al. 2017. “Habitat‐Specific Patterns and Drivers of Bacterial β‐Diversity in China's Drylands.” ISME Journal 11: 1345–1358. 10.1038/ismej.2017.11. 28282041 PMC5437346

[ece371604-bib-0074] Wetzels, M. , G. Odekerken‐Schröder , and C. v. Oppen . 2009. “Using PLS Path Modeling for Assessing Hierarchical Construct Models: Guidelines and Empirical Illustration.” MIS Quarterly 33: 177–195.

[ece371604-bib-0075] Włodarska‐Kowalczuk, M. , J. Pawłowska , and M. Zajączkowski . 2013. “Do Foraminifera Mirror Diversity and Distribution Patterns of Macrobenthic Fauna in an Arctic Glacial Fjord?” Marine Micropaleontology 103: 30–39. 10.1016/j.marmicro.2013.07.002.

[ece371604-bib-0076] Xie, Y. 1979. “Underwater Topography of Xisha Island Sea Area [in Chinese].” Marine Science Bulletin 3: 24–33.

[ece371604-bib-0077] Yu, M. , and Y. Sun . 2024. “Decoding the Popularity of TV Series: A Network Analysis Perspective.” 2024 4th International Conference on Computer Communication and Artificial Intelligence (CCAI). 10.1109/CCAI61966.2024.

[ece371604-bib-0078] Zhao, Q. 2020. “The Sedimentary Research About Reef Carbonatite in Xisha Islands Waters.” PhD Thesis, Qingdao, China. Institute of Oceanology, Chinese Academy of Sciences.

[ece371604-bib-0079] Zhou, J. , and D. Ning . 2017. “Stochastic Community Assembly: Does It Matter in Microbial Ecology?” Microbiology and Molecular Biology Reviews 81, no. 4: e00002‐17. 10.1128/mmbr.00002-17. 29021219 PMC5706748

[ece371604-bib-0100] Zhu, Y. , and R. Shen . 2021. China Soil Microbiome Initiative. Zhejiang University Press.

