# Supplementary material for: Drift Drives Foraminiferal Community Assembly on a Carbonate Platform
Source: Ecol Evol. 2025 Jun 18;15(6):e71604. doi: 10.1002/ece3.71604 (PMC12174969; doi:10.1002/ece3.71604)
Supplement: Supplementary file 1 — Appendix S1. [file ECE3-15-e71604-s001.pdf]

Supplemental Information for:

Drift drives foraminiferal community assembly on a carbonate platform

Tao Li, Bo Li, Ziya Lin, Wei Xie, Chupeng Yang

Table of Contents:

**Environmental DNA extraction, PCR amplification and high-throughput sequencing**	Page 1
**Figure S1**	Page 2
**Figure S2**	Page 3
**Figure S3**	Page 4
**Table S1**	Page 5
**Table S2**	Page 6
**Table S3**	A separated excel file
**Table S4**	Page 7
**Table S5**	Page 8
**Table S6**	Page 9

Environmental DNA extraction, PCR amplification and high-throughput sequencing

eDNA was extracted from sediment via the FastDNA® Spin Kit for Soil (MP bio, USA) according to the manufacturer's instructions, whereas the lysis time was prolonged to 40 minutes to improve the efficiency of DNA extraction. Three replicate samples were taken at every sampling site for DNA extraction. The quality and concentration of the extracted DNA were assessed and quantified via a NanoDrop 2000 spectrophotometer (Thermo Fisher Scientific, Wilmington, DE, USA). The hypervariable region of the nuclear 18S rRNA gene (37 + 41f) was amplified via PCR using foraminiferal–specific primers (forward F1 5′–AAGGGCACCACAAGAACGC–3′ and reverse 17–5′–CGGTCACGTTCGTTGC–3′) (Frontalini *et al.* 2020), which produced sequences ranging from 230 to 380 bp. Negative and positive controls were included to prevent possible contamination. The PCR was performed in a 20 μL mixture containing 4 μL of 5 × TransStart FastPfu buffer, 2 μL of 2.5 mM dNTPs, 0.8 μL of each primer at 5 μM, 0.4 μL of TransStart FastPfu DNA Polymerase, and 1μL (~10 ng) of the extracted DNA. The PCR conditions were as follows: initial denaturation at 95 °C for 30 seconds, followed by 27 cycles of denaturation at 95 °C for 30 seconds, annealing at 55 °C for 30 seconds, and elongation at 72 °C for 45 seconds. The PCR products were purified via the AxyPrep DNA Gel Extraction Kit (Axygen Biosciences, USA) and further quantified via a Quantus™ Fluorometer (Promega, USA). The NEXTFLEX Rapid DNA–Seq Kit (Bioo Scientific, USA) was used to generate libraries, and the Illumina PE300 platform was used for sequencing; both methods were conducted by Shanghai Majorbio Pharm Technology Co. Ltd.

References:

Frontalini, F., T. Cordier, E. Balassi, E. Armynot du Chatelet, et al. 2020. "Benthic foraminiferal metabarcoding and morphology–based assessment around three offshore gas platforms: Congruence and complementarity." *Environment International* 144: 106049. https://doi.org/10.1016/j.envint.2020.106049.

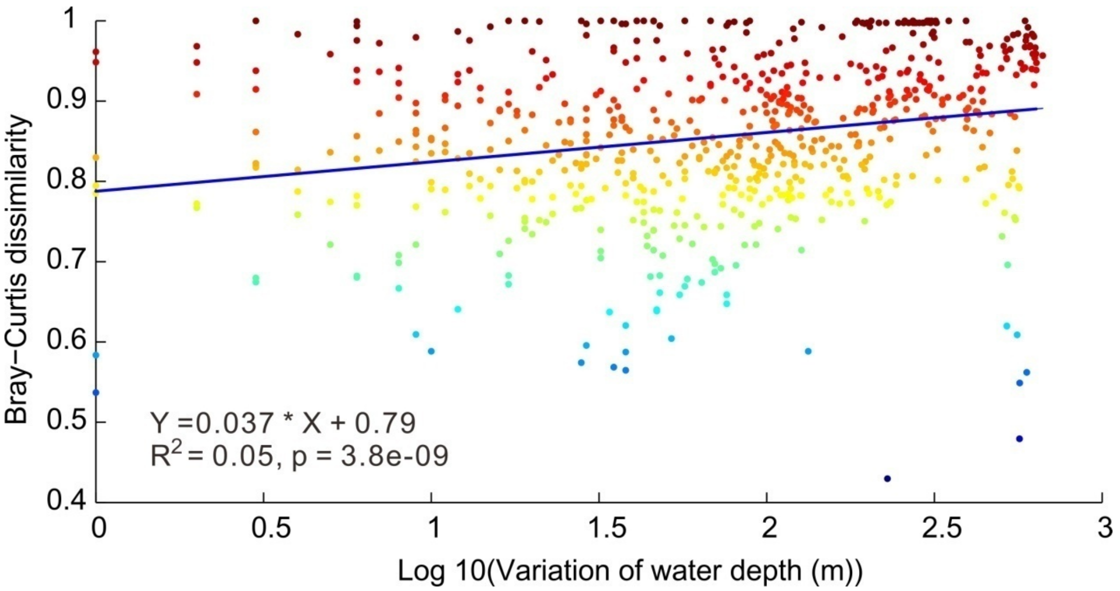

Figure S1. The correlation between the Bray-Curtis dissimilarity and the variation of water depth.

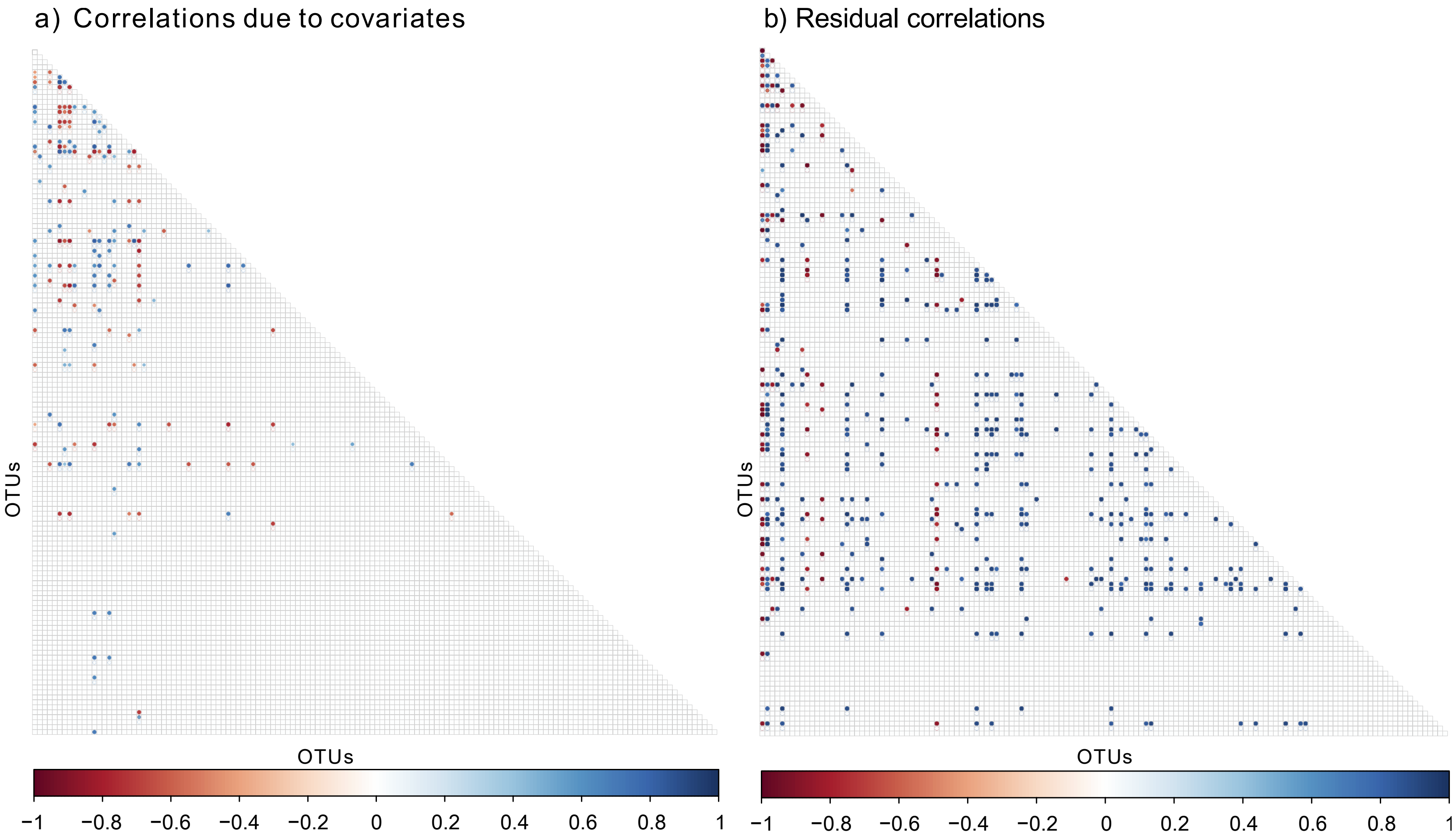

Figure S2. Plots of the correlations between OTUs due to the environmental covariates (a) and residual correlations (b). Only the significant correlations have been plotted. Negative and positive correlations are represented by red and blue, respectively.

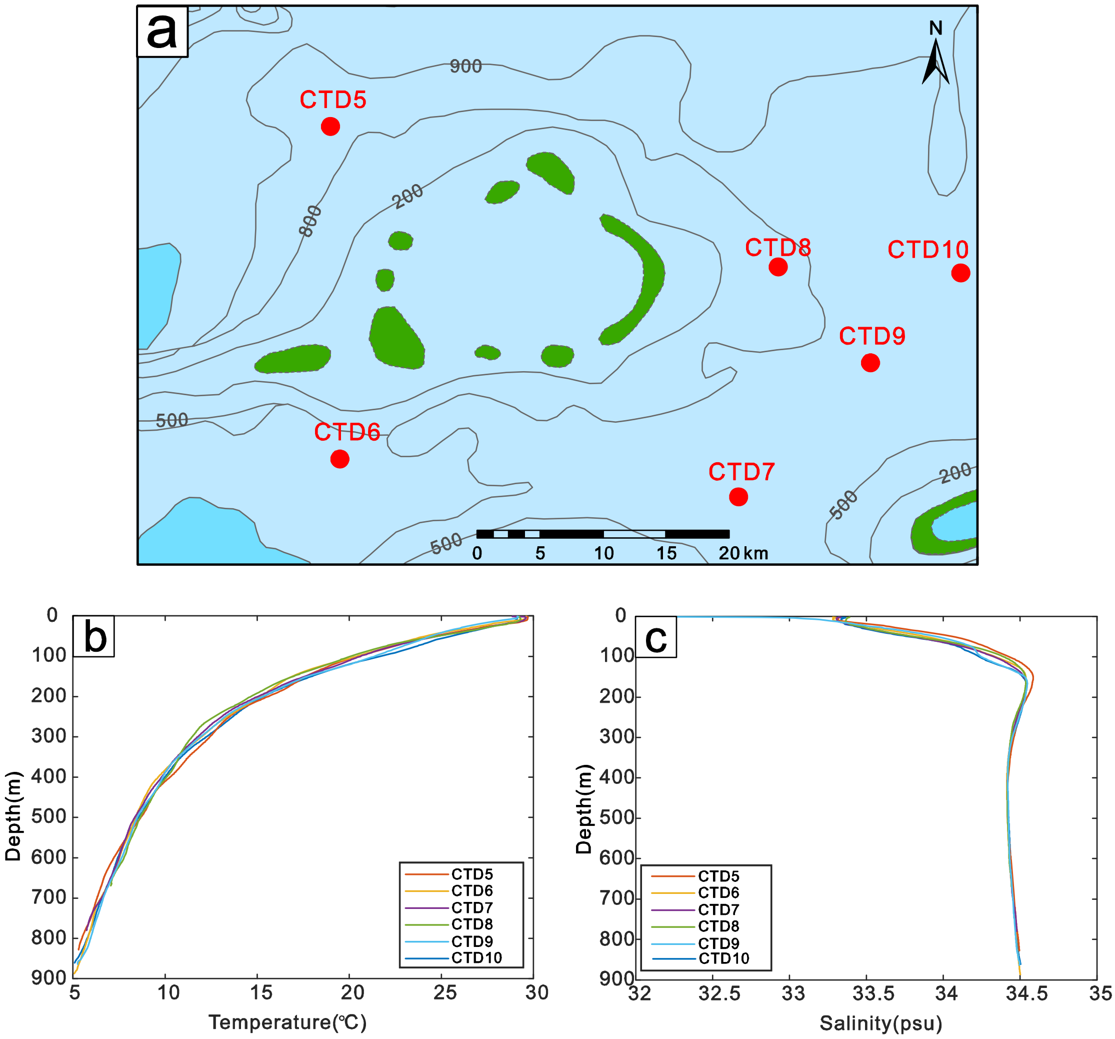

Figure S3. Map of CTD (conductivity, temperature, and depth) stations (a) and parameters such as temperature (b) and salinity (c) showing in the CTD stations.

Table S1. The physicochemical characteristics of 37 sediment samples.

Sites	Grain sizes (%)			pH	$CaCO_3$ (%)	OC (%)	LOI (%)	Heavy metals (mg kg^{-1})						
	Sand	Silt	Clay					Co	Ni	Cu	Zn	V	Cr	Pb
2	11.29	71.71	17.00	7.612	55.35	1.01	32.80	9.35	48.5	17.9	66.0	53.9	25.7	17.7
4	17.46	64.50	18.03	7.595	6 6.21	0.80	36.53	6.18	25.1	12.5	39.6	35.2	27.4	13.8
5	12.62	64.33	23.06	7.574	56.60	0.91	33.23	8.79	45.5	17.4	62.2	48.9	34.5	16.0
6	32.71	56.33	10.95	7.585	77.73	0.78	40.01	3.63	12.6	8.1	23.9	21.4	n.d.	10.7
7	13.45	63.75	22.79	7.552	57.55	0.91	34.11	8.23	36.5	15.7	53.1	46.7	30.9	14.8
8	33.10	54.49	12.41	7.535	67.08	0.70	36.10	6.65	27.2	11.6	40.1	35.1	17.7	14.9
10	17.31	63.86	18.85	7.607	68.75	0.73	37.32	6.26	23.7	11.0	47.2	30.4	35.3	12.6
11	11.75	66.01	22.25	7.556	54.70	1.00	33.68	8.42	42.3	17.4	67.5	50.1	24.6	16.5
13	35.19	47.81	16.98	7.585	72.35	0.59	37.72	5.63	22.0	11.1	32.7	30.9	14.0	13.7
14	17.19	60.51	22.30	7.587	57.41	0.84	33.69	7.49	33.8	16.1	54.9	47.6	38.4	16.3
15	13.34	63.62	23.05	7.541	56.30	0.90	32.97	7.78	43.5	17.5	60.9	51.2	35.3	17.3
16	11.69	67.86	20.45	7.667	56.22	0.86	33.09	7.58	30.9	15.1	54.5	51.0	35.3	16.6
18	19.88	61.90	18.22	7.603	67.39	0.80	37.71	5.85	20.9	11.4	37.3	31.9	n.d.	13.1
19	7.98	68.39	23.63	7.582	57.26	0.93	33.92	9.83	38.6	17.0	56.5	45.7	38.8	17.5
20	17.44	64.69	17.87	7.724	63.57	0.80	35.01	7.20	28.5	13.2	45.7	37.4	21.5	14.6
22	31.47	55.05	13.47	7.623	64.42	0.66	34.51	7.08	28.7	12.6	45.7	39.4	20.0	16.0
23	41.69	46.28	12.04	7.625	71.64	0.56	36.91	7.23	24.0	9.7	38.9	32.1	25.2	15.1
24	27.31	55.51	17.17	7.645	71.78	0.61	37.98	6.06	20.1	9.2	32.6	29.7	18.0	13.1
25	9.22	65.04	25.73	7.559	44.28	0.98	29.26	10.30	51.3	19.8	74.2	65.8	50.3	18.3
26	59.82	17.67	3.64	7.859	85.20	0.33	43.09	n.d.	4.1	2.0	10.0	8.3	n.d.	6.1
27	15.59	67.19	17.21	7.621	63.48	0.85	35.89	7.63	28.3	12.8	44.7	36.6	26.1	12.3
31	36.16	46.16	17.68	7.596	69.07	0.60	37.18	5.62	22.1	11.4	39.5	38.6	25.7	13.6
32	27.98	59.46	12.56	7.662	67.18	0.72	36.57	6.09	22.1	11.3	38.6	35.1	19.5	14.9
33	10.11	72.46	17.42	7.576	45.46	0.98	28.95	11.10	48.5	20.1	72.4	63.1	56.6	19.4
34	40.86	48.23	10.91	7.676	76.54	0.61	39.80	4.65	16.1	8.1	28.1	21.6	14.7	11.9
39	12.48	70.98	16.55	7.854	50.60	0.89	31.31	8.92	42.7	18.9	65.3	58.5	49.2	16.4
40	13.28	69.44	17.27	7.627	51.05	0.98	31.68	8.74	38.8	18.6	62.1	56.8	45.2	17.6
42	14.60	69.36	16.03	7.626	51.96	0.95	32.25	8.86	37.2	17.8	69.9	54.7	32.0	15.0
43	16.12	65.91	17.97	7.598	49.37	0.91	30.95	11.30	44.1	19.9	67.6	60.0	44.1	18.1
44	70.30	25.38	4.32	7.901	87.15	0.33	43.84	n.d.	4.1	2.1	21.3	7.5	n.d.	6.6
45	32.79	56.30	10.92	7.589	72.28	0.77	39.12	5.66	19.1	11.2	31.1	29.6	n.d.	10.8
50	65.09	28.58	5.96	7.697	87.23	0.42	43.70	n.d.	4.4	1.5	9.5	7.7	n.d.	5.5
51	10.24	73.18	16.58	7.531	43.79	1.02	29.25	12.50	63.1	23.0	81.7	66.1	52.1	17.4
52	65.59	24.23	5.08	7.599	83.18	0.41	42.11	4.31	12.1	3.0	18.0	18.3	n.d.	10.2
54	11.32	70.21	18.47	7.482	40.66	1.00	27.95	12.80	79.8	24.3	86.9	73.0	47.4	17.9
55	15.33	68.41	16.25	7.535	60.34	0.90	34.66	8.79	34.5	15.5	53.1	45.5	24.3	15.2
56	68.86	24.92	4.86	7.844	86.81	0.36	42.86	n.d.	6.1	2.4	13.2	9.7	n.d.	7.0

n.d., not detected

Table S2. Geoaccumulation index (Igeo) values of heavy metals in 37 sediment samples.

Sites	Co	Ni	Cu	Zn	V	Cr	Pb
2	–0.896	0.798	–0.261	–0.241	–0.561	–1.031	–0.527
4	–1.493	–0.153	–0.779	–0.978	–1.175	–0.938	–0.886
5	–0.985	0.706	–0.302	–0.327	–0.701	–0.606	–0.672
6	–2.261	–1.147	–1.401	–1.706	–1.893	NA	–1.253
7	–1.080	0.388	–0.450	–0.555	–0.768	–0.765	–0.785
8	–1.388	–0.037	–0.887	–0.960	–1.179	–1.569	–0.775
10	–1.475	–0.235	–0.963	–0.725	–1.387	–0.573	–1.017
11	–1.047	0.600	–0.302	–0.209	–0.666	–1.094	–0.628
13	–1.628	–0.343	–0.950	–1.254	–1.363	–1.907	–0.896
14	–1.216	0.277	–0.414	–0.507	–0.740	–0.451	–0.646
15	–1.161	0.641	–0.294	–0.357	–0.635	–0.573	–0.560
16	–1.199	0.147	–0.506	–0.517	–0.640	–0.573	–0.619
18	–1.573	–0.417	–0.912	–1.064	–1.317	NA	–0.961
19	–0.824	0.468	–0.335	–0.465	–0.799	–0.436	–0.543
20	–1.273	0.031	–0.700	–0.771	–1.088	–1.288	–0.805
22	–1.297	0.041	–0.768	–0.771	–1.013	–1.392	–0.672
23	–1.267	–0.217	–1.148	–1.004	–1.308	–1.059	–0.756
24	–1.522	–0.473	–1.218	–1.259	–1.420	–1.544	–0.961
25	–0.756	0.879	–0.115	–0.072	–0.273	–0.062	–0.479
26	NA	–2.763	–3.423	–2.963	–3.265	NA	–2.073
27	–1.189	0.021	–0.745	–0.803	–1.119	–1.008	–1.052
31	–1.630	–0.336	–0.912	–0.982	–1.042	–1.031	–0.907
32	–1.515	–0.336	–0.925	–1.015	–1.179	–1.429	–0.775
33	–0.649	0.798	–0.094	–0.107	–0.333	0.108	–0.394
34	–1.904	–0.793	–1.403	–1.473	–1.880	–1.837	–1.100
39	–0.964	0.614	–0.183	–0.256	–0.443	–0.094	–0.637
40	–0.993	0.476	–0.206	–0.329	–0.485	–0.216	–0.535
42	–0.974	0.415	–0.269	–0.158	–0.539	–0.714	–0.766
43	–0.623	0.661	–0.108	–0.206	–0.406	–0.252	–0.495
44	NA	–2.774	–3.380	–1.873	–3.410	NA	–1.943
45	–1.620	–0.547	–0.937	–1.327	–1.425	NA	–1.239
50	NA	–2.671	–3.887	–3.041	–3.361	NA	–2.224
51	–0.477	1.177	0.101	0.067	–0.266	–0.011	–0.551
52	–2.013	–1.205	–2.848	–2.115	–2.119	NA	–1.322
54	–0.443	1.516	0.180	0.156	–0.123	–0.147	–0.511
55	–0.985	0.306	–0.469	–0.555	–0.805	–1.111	–0.746
56	NA	–2.196	–3.154	–2.563	–3.038	NA	–1.869
Mininum	–2.261	–2.774	–3.887	–3.041	–3.410	–1.907	–2.224
Maximum	–0.443	1.516	0.180	0.156	–0.123	0.108	–0.394
Average	–1.229	–0.178	–1.002	–0.927	–1.223	–0.819	–0.928

NA, not available

Table S4. Alfa-diversities of 37 benthic foraminiferal communities.

Sites	ACE	Shannon	Simpson
2	56	4.458	0.895
4	54	4.117	0.882
5	103	4.973	0.918
6	44	4.197	0.924
7	62	3.863	0.792
8	85	5.122	0.952
10	54	3.442	0.738
11	83	5.064	0.934
13	74	4.528	0.902
14	45	3.855	0.878
15	71	4.765	0.920
16	76	4.905	0.944
18	57	4.379	0.918
19	70	5.102	0.944
20	78	5.160	0.945
22	58	4.337	0.865
23	64	4.851	0.937
24	74	5.169	0.954
25	59	3.598	0.769
26	74	4.463	0.899
27	44	2.694	0.602
31	14	1.778	0.612
32	129	5.230	0.938
33	48	2.282	0.512
34	111	5.565	0.952
39	24	3.334	0.834
40	68	4.670	0.924
42	70	4.855	0.933
43	80	3.667	0.827
44	55	4.142	0.896
45	45	3.581	0.800
50	7	0.341	0.079
51	70	5.092	0.949
52	70	4.333	0.890
54	36	2.724	0.668
55	44	4.593	0.938
56	18	3.236	0.858

Table S5. The topological properties of 37 sample sub-networks.

Sites	Average degree	Average distance	Betweenness centrality	Degree centrality	Eigenvector centrality	Density	Transitivity
2	1.120	1.767	0.038	0.120	0.913	0.047	0.231
4	0.846	2.037	0.038	0.086	0.917	0.034	0.000
5	1.429	1.815	0.017	0.111	0.915	0.035	0.536
6	0.933	1.538	0.030	0.148	0.866	0.067	0.375
7	1.600	2.036	0.026	0.083	0.918	0.055	0.441
8	1.517	2.417	0.063	0.124	0.851	0.054	0.349
10	1.143	2.241	0.213	0.220	0.835	0.088	0.000
11	1.000	1.400	0.008	0.077	0.935	0.026	0.625
13	1.167	2.171	0.054	0.123	0.877	0.051	0.333
14	1.222	2.094	0.109	0.163	0.862	0.072	0.231
15	2.643	2.244	0.133	0.235	0.762	0.098	0.420
16	1.091	2.556	0.068	0.122	0.901	0.034	0.222
18	0.960	1.654	0.031	0.127	0.940	0.040	0.000
19	1.758	2.250	0.091	0.133	0.867	0.055	0.356
20	1.697	2.230	0.040	0.134	0.854	0.053	0.500
22	2.300	1.558	0.041	0.195	0.780	0.121	0.609
23	3.000	2.481	0.233	0.310	0.779	0.103	0.462
24	2.108	3.065	0.213	0.164	0.847	0.059	0.402
25	1.238	2.122	0.157	0.238	0.881	0.062	0.136
26	2.400	2.509	0.123	0.224	0.842	0.071	0.652
27	0.889	1.429	0.036	0.183	0.916	0.052	0.333
31	0.500	1.000	0.000	0.071	0.667	0.071	NA
32	1.434	1.597	0.005	0.049	0.950	0.028	0.577
33	0.778	1.125	0.007	0.072	0.974	0.046	0.000
34	3.000	2.972	0.189	0.213	0.850	0.064	0.422
39	2.250	1.400	0.286	0.393	0.623	0.321	0.714
40	1.467	2.416	0.195	0.225	0.862	0.051	0.120
42	1.355	2.032	0.037	0.122	0.865	0.045	0.300
43	0.727	1.200	0.002	0.071	0.950	0.023	0.600
44	2.769	1.750	0.163	0.186	0.597	0.231	0.714
45	0.500	1.333	0.009	0.100	0.970	0.033	0.000
50	0.000	NA	NA	0.000	NA	0.000	NA
51	1.259	1.977	0.037	0.105	0.873	0.048	0.346
52	0.833	1.231	0.004	0.051	0.955	0.036	0.500
54	0.364	1.333	0.022	0.164	0.954	0.036	0.000
55	1.111	2.000	0.037	0.052	0.844	0.065	0.000
56	1.636	1.563	0.078	0.136	0.837	0.164	0.545

NA, not available

Table S6. Pearson correlation between the topological properties of sample sub－networks and physiochemical parameters and community α–diversities.
* for $p < 0.05$, ** for $p < 0.01$

	Average degree	Average path distance	Betwenness centrality	Degree centrality	Eigenvector centrality	Density	Transitivity
Sand	**0.383***	0.016	0.128	0.059	–0.305	**0.321***	**0.436***
Silt	**–0.402***	–0.086	–0.146	–0.084	0.276	–0.271	**–0.458***
Clay	–0.268	0.101	–0.048	–0.029	0.248	**–0.301***	**–0.340***
pH	**0.521****	0.071	**0.432****	**0.407***	**–0.608****	**0.688****	**0.554****
$CaCO_3$	**0.359***	0.168	0.132	0.016	–0.247	0.212	**0.324***
OC	**–0.465****	–0.178	–0.242	–0.139	**0.354***	**–0.331***	**–0.429***
LOI	**0.321***	0.148	0.112	–0.013	–0.223	0.195	0.308
Co	–0.385	–0.166	–0.182	–0.095	0.333	–0.316	**–0.353***
Ni	–0.345	–0.209	–0.165	–0.021	0.243	–0.204	–0.295
Cu	–0.370	–0.171	–0.157	–0.037	0.253	–0.228	**–0.342***
Zn	–0.319	–0.184	–0.110	–0.003	0.180	–0.165	–0.285
V	–0.362	–0.183	–0.131	–0.015	0.244	–0.203	**–0.318***
Cr	–0.164	–0.014	0.137	0.164	0.064	–0.045	–0.244
Pb	–0.248	–0.057	–0.098	–0.038	0.246	–0.269	–0.287
ACE	0.227	**0.371***	–0.123	–0.267	0.233	**–0.456****	0.305
Shannon	**0.426***	**0.611****	0.075	–0.106	–0.100	–0.226	0.305
Simpson	**0.404***	**0.509****	0.134	–0.028	–0.255	–0.009	**0.350***
